# 3D printed ventilation tubes and their effect on biological models

**DOI:** 10.1186/s41205-024-00225-y

**Published:** 2024-07-02

**Authors:** Luis Humberto Govea-Camacho, Irma Yolanda Castillo-López, Sergio Alejandro Carbajal-Castillo, Alejandro Gonzalez-Ojeda, Gabino Cervantes-Guevara, Enrique Cervantes-Pérez, Sol Ramírez-Ochoa, Sergio Jiram Vázquez-Sánchez, Gonzalo Delgado-Hernández, Jaime Alberto Tavares-Ortega, Samantha Emily González-Muñoz, Clotilde Fuentes-Orozco

**Affiliations:** 1 Departamento de Fisiología, Centro Universitario de Ciencias de la Salud, Universidad de Guadalajara, Guadalajara, Jalisco Mexico; 2 Servicio de Otorrinolaringología, Instituto Mexicano del Seguro Social (IMSS), Hospital General Regional no. 46. Av. Lázaro Cárdenas No. 2063, Guadalajara, Jalisco Mexico; 3 Unidad de Investigación Biomédica 02, Hospital de Especialidades, Centro Médico Nacional de Occidente, Guadalajara, Jalisco Mexico; 4 Departamento de Bienestar y Desarrollo Sustentable, Centro Universitario del Norte, Universidad de Guadalajara, Carretera Federal No. 23, Km. 191, Colotlán, Jalisco C.P. 46200 Mexico; 5 Departamento de Medicina Interna, Centro Universitario de Ciencias de la Salud, Hospital Civil de Guadalajara Fray Antonio Alcalde, Universidad de Guadalajara, Guadalajara, Jalisco Mexico; 6 Departamento de Otorrinolaringología y Cirugía de Cabeza y Cuello, Hospital de Especialidades, Centro Médico Nacional de Occidente, Guadalajara, México

**Keywords:** Implants, 3D printing, Ventilation tubes, Acute otitis media

## Abstract

**Background:**

Acute otitis media (AOM) causes inflammation and hearing loss. Ventilation tubes are key in treatment. 3D printing improves prostheses in otorhinolaryngology, offering precision and greater adaptability.

**Materials and methods:**

An experimental study was conducted with Wistar rats from July to December 2020. 3D tympanostomy tube models were designed, with technical specifications and tests performed on inexpensive 3D printers. The tympanostomy tube was inserted endoscopically.

**Results:**

Procedures were performed on five rats with implants in both ears. Pre-intervention pathologies, such as atical retraction and glue ear, were found. The PLA-printed tympanostomy tube showed improvement after adjustments. Histopathological results revealed significant middle and inner ear damage.

**Conclusion:**

In our study, the design and 3D printing of implants fulfilled the desired functions when modified, with a height of 5 mm. Complications included PLA degradation and ear damage. There were no adverse events during observation, highlighting the need for further research on 3D-printed implants.

## Background

Acute otitis media (AOM) is a disease characterized by the presence of fluid in the middle ear, accompanied by the sudden appearance of signs and symptoms of inflammation in this area. The presence of fluid in the middle ear can cause significant discomfort and affect hearing, resulting in a hearing loss of 25 to 30 dB [1, 2].

Treatment for acute otitis media includes pain management, observation, or antibiotics. Tympanostomy tubes are considered for children experiencing three or more episodes within six months or four episodes within a year, with at least one episode in the preceding six months [3]. The placement of the tubes, a key treatment for otitis media, addresses hearing impairment and damage to the tympanic membrane and is the intervention of choice [4].

Three materials—fluoroplastic, silicone elastomer, and metal—are utilized alongside two fundamental designs, short-term and long-term, in the majority of tympanostomy tubes. [5].

In 1986, 3D printing debuted as additive manufacturing, revolutionizing production with rapid prototyping and innovative three-dimensional technology [6]. 3D model printing allows a system to create 3D objects with information from digital databases [7]. These printed models can mimic all the shapes needed in real patient anatomies, helping to improve patient care and surgical outcomes [8].

3D printing in medicine has mainly focused on non-biological static structures, such as structural or space-filling prostheses [9].

In terms of otorhinolaryngology, the use of prosthesis or implants is frequent; of these, tympanostomy tubes are the most commonly used, and with greater innovation. These have been updated with a retractable blade as a safety element, as well as the incorporation of a “backstop” proximal to the tip of the device to minimize the risk of unintentional over-insertion; during their innovation, 3D printed prototype devices were obtained for greater accessibility [10, 11].

Among the advantages this technology offers is the opportunity to create 3D-printed medical devices in small sizes to suit the wide range of needs of the pediatric population. Additionally, the greater precision of 3D-printed surgical guides has been proven to reduce complications and intervention time [12, 13].

### Study hypothesis

Polylactic acid (PLA) is a biocompatible and biodegradable polymer that has been used in subdermal implants for prolonged drug release. Similarly, it has been used in the field of surgery through plates and screws that degrade over time, thus avoiding re-interventions for material removal.

Our objective was to assess the auditory effects and safety of using tympanostomy tubes printed in polylactic acid and placed in the tympanic membrane.

## Materials and methods

An experimental study was carried out from July 2020 to December 2020 using Wistar strain rats from the Laboratory of Breeding and Reproduction of Animals for Experimentation at the University Center of Health Sciences (biotherium).

### Population

Apparently, healthy Wistar strain rats were included. Rats with tympanic perforations before tympanostomy tube placement and those with external or middle ear malformations were excluded.

### Phases

It was divided into two phases; in the first phase, ventilation tube models were designed for 3D printing. In the second phase, an experimental study was carried out with animals from the laboratory of breeding and reproduction of animals for experimentation.

The digital design of the tympanostomy tube was made taking into account the following characteristics: internal diameter 1.1 mm, external diameter 2.2 mm, and length 1.8 mm. At its upper end the tube has a 0.4 mm flange that protrudes from the cylinder, at the lower end the flange protrudes from the cylinder by 0.2 mm. In this way, its design will be similar to the Shepherd tubes. The tube was packed in surgical paper and sent to steam sterilization according to the conventional technique.

### Implant

Solid Works 2018 software was used to design the 3D part, and for the transfer to the printer, Ultimaker Cura 4.1 and Cura 14.01 software were used to generate the G-codes. Tests were performed with both software.

**Fig. 1:**
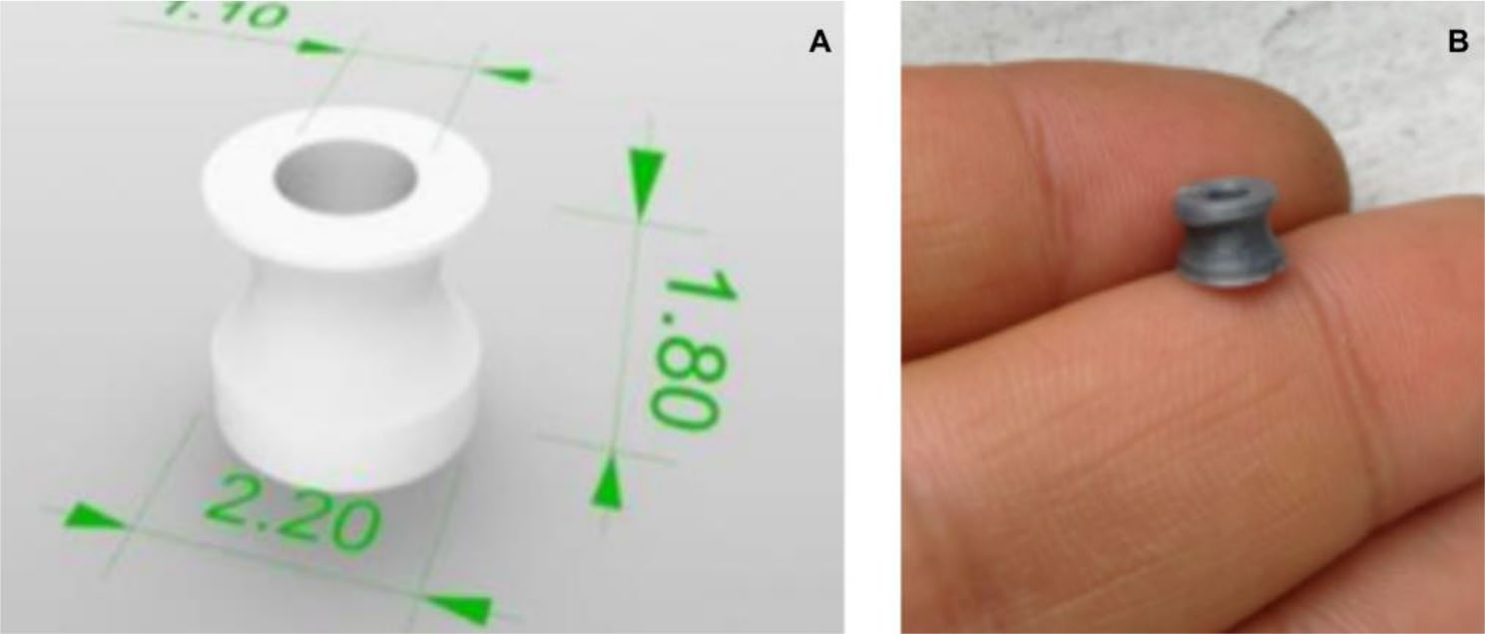
(**A**) Model of a tympanostomy tube. (**B**) PLA tympanostomy tube with a height of 5 mm

The models were printed on material extrusion model printers of the ANET A8, Ender 3, and TARANTULA I3 models, which are homemade 3D printers of the economic range. A resolution quality of 0.05 mm was used for 100% density. The printed model was not very stable and with low stiffness, making it easily collapsible. New tests were carried out with the 0.2 mm and 0.4 mm extruder tip, with which a stable and firm model was achieved, with adequate stiffness when growing it at 278%, leaving it with a height of 5 mm (Fig. 1).

### Surgical procedure

The specimens were identified employing an adhesive bracelet with the numbers 1,2,3,4 and 5. Before anesthesia, the rats were weighed to calculate the anesthetic dose. Anesthesia and analgesia with ketamine 70 mg/kg b.w. and xylazine 15 mg/k b.w. infiltrated intraperitoneally. EOA was performed with the TITAN equipment of interacoustic using the DPOAES440 6 Hz module (500-8k Hz maximum 30 Db). It was considered positive when there was a replica at the scanned frequency (check mark) and negative when no replica was obtained (cross mark). Endoscopic exploration of the tympanic membrane with Storz 1.7 mm endoscopic lens. The PLA tympanostomy tube was inserted under endoscopic vision after myringotomy in the posteroinferior quadrant, leaving a portion protruding into the external auditory canal. Since it was only possible to insert PLA tympanostomy tubes in 5 ears out of the 10 possible ears, we divided the ears into two groups: (1) With PLA insertion and (2) Without PLA insertion. Three months later, after anesthesia and analgesia with ketamine 70 mg/kg body weight and xylazine 15 mg/kg body weight infiltrated intraperitoneally, endoscopic exploration of the ears was performed to verify the permanence of the PLA tympanostomy tube or its absorption. EOA is taken again with the TITAN equipment of interacoustic using the DPOAES440 6 Hz module (500-8k Hz maximum 30 Db). Subsequently, a lethal dose of sodium pentobarbital (90–120 mg/kg) is administered by intraperitoneal injection to each mouse for sacrifice of the experimental specimens.

### Statistical analysis

A database was used with the use of Excel Software and Statistical Package for Social Sciences (SPSS), version 20 for Windows, a database was created and processed. The results were presented with descriptive statistics analysis. Statistical inference tests were performed to contrast the specific objectives through Fisher’s Chi-Square or Exact Chi-Square test and Student’s T-test.

### Ethical considerations

All procedures were carried out following the institutional guidelines of the laboratory for breeding and reproduction of animals for experimentation at the Centro Universitario de Ciencias de la Salud of the University of Guadalajara, which are in accordance with those approved by the Federal Law of Animal Health, NOM−062-ZOO−1999, and NOM−033-ZOO−1995.

## Results

**Fig. 2:**
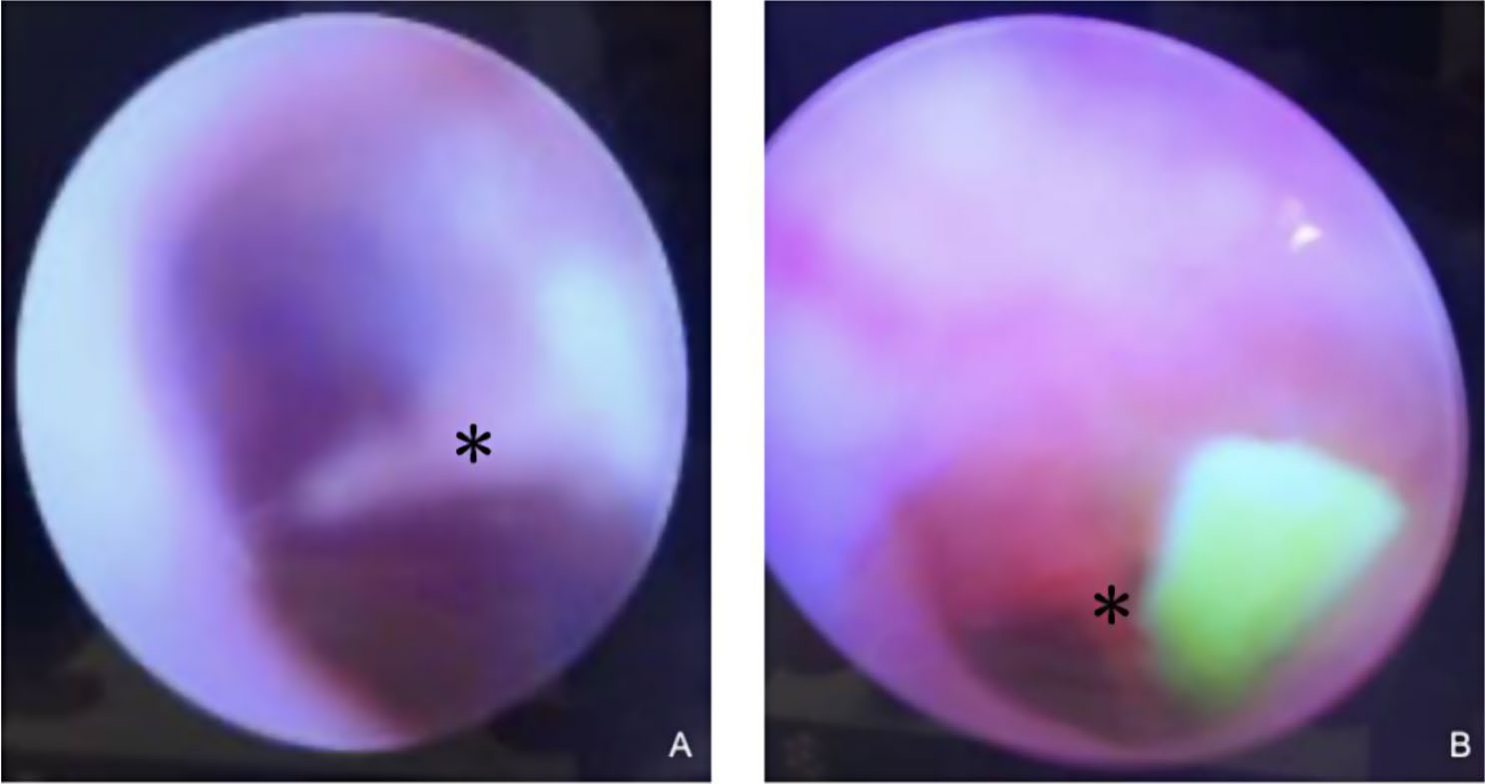
(**A**) Endoscopic view of the normal anatomy of the tympanic membrane previous to the procedure. Malleus is identified by an asterisk. (**B**) Tympanostomy tube placed in biological model, the tympanic membrane is seen in-depth. Malleus is identified by an asterisk

Five rats were included, all females, where implants were placed in both right and left ears for a total of 5 ears, in the rest the procedure could not be performed because an adhesive tympanic membrane was found during the exploration, this group was taken as control (Fig. 2).

**Fig. 3:**
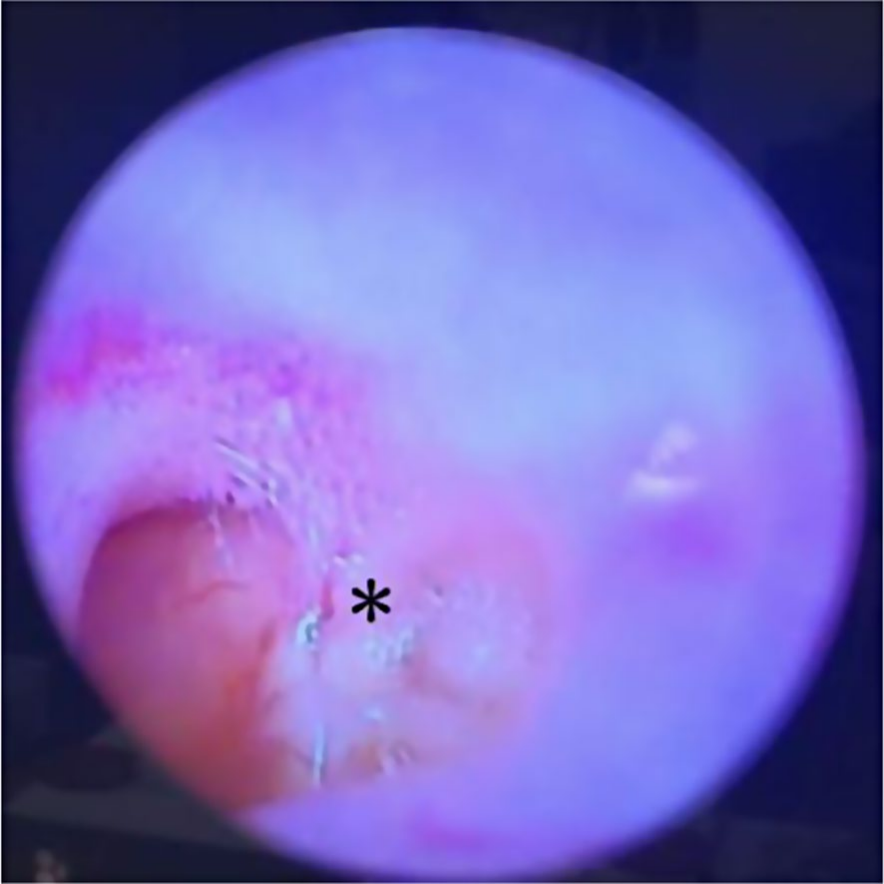
Adhesive tympanic membrane in the left ear of specimen #3, important retraction skeletonizing malleus manubrium identified by an asterisk

Of the five in which the intervention was achieved, two ears were found with normal tympanic membrane and three ears with atical retraction, corresponding to specimens #2 bilateral, #1 bilateral, and #3 right ear (Fig. 3).

### General characteristics of experimental ears

A prevalence of pre-intervention middle ear pathology was found in 80% of the specimens. Of these, 30% were of the attic retraction type, and 50% were of the adhesive otitis type. The remaining 20% were clinically unaltered.

Preintervention DPOAEs (otoacoustic emission distortion products) were performed. They were found to be present in 100% of Group 1 and 0% of Group 2. (Table 1). Ears with absent emissions corresponded to ears with adhesive tympanic membranes. At post-intervention assessment, otoacoustic emissions were absent in all the ears examined (100%).

**Table 1:** Pre and postoperative otoacoustic emissions

	Study specimen			Pre-operative	Post-operative
Number	Ear	Group	PLA insertion	Otoacoustic emissions	Otoacoustic emissions
1	R	1	Yes	Present	Absent
1	L	1	Yes	Present	Absent
2	R	1	Yes	Present	Absent
2	L	1	Yes	Present	Absent
3	R	1	Yes	Present	Absent
3	L	2	No	Absent	Absent
4	R	2	No	Absent	Absent
4	L	2	No	Absent	Absent
5	R	2	No	Absent	Absent
5	L	2	No	Absent	Absent

R: right; L: left

**Fig. 4:**
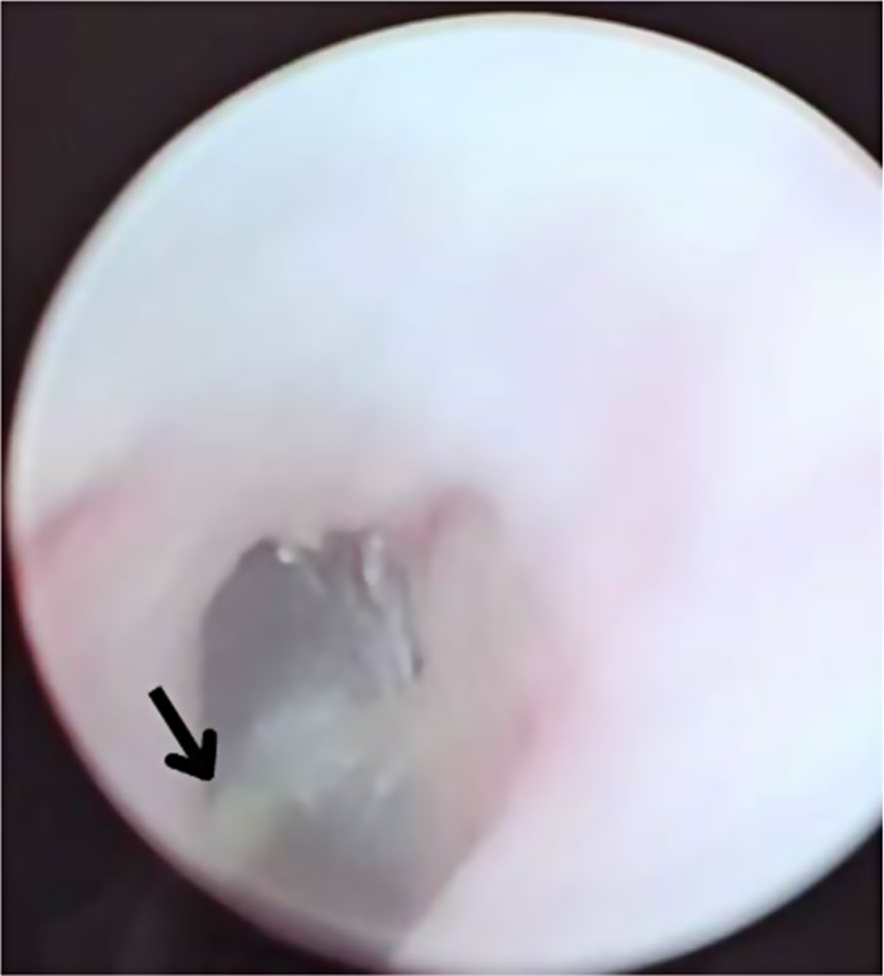
Endoscopic view. The black arrow shows the residual PLA in the middle ear seen throw the translucent tympanic membrane at a 3-month evaluation

Tympanostomy tube biodegradation time, tympanic membrane repair, and associated complications: At the first evaluation, at 3 months, remnants of PLA were observed in one ear, in the rest there was no evidence of PLA with completely repaired tympanic membrane (Fig. 4). During the observation period, there were no adverse events such as otorrhea, otorrhagia, granulomas, or other lesions, and all specimens survived (Table 2).

### Histopathological results

Middle Ear: We observed minimal to mild damage to the epithelial layer of the tympanic membrane and inflammatory damage in the middle ear in 80% of Group 1, whereas none of the ears in Group 2 had damage.

**Fig. 5:**
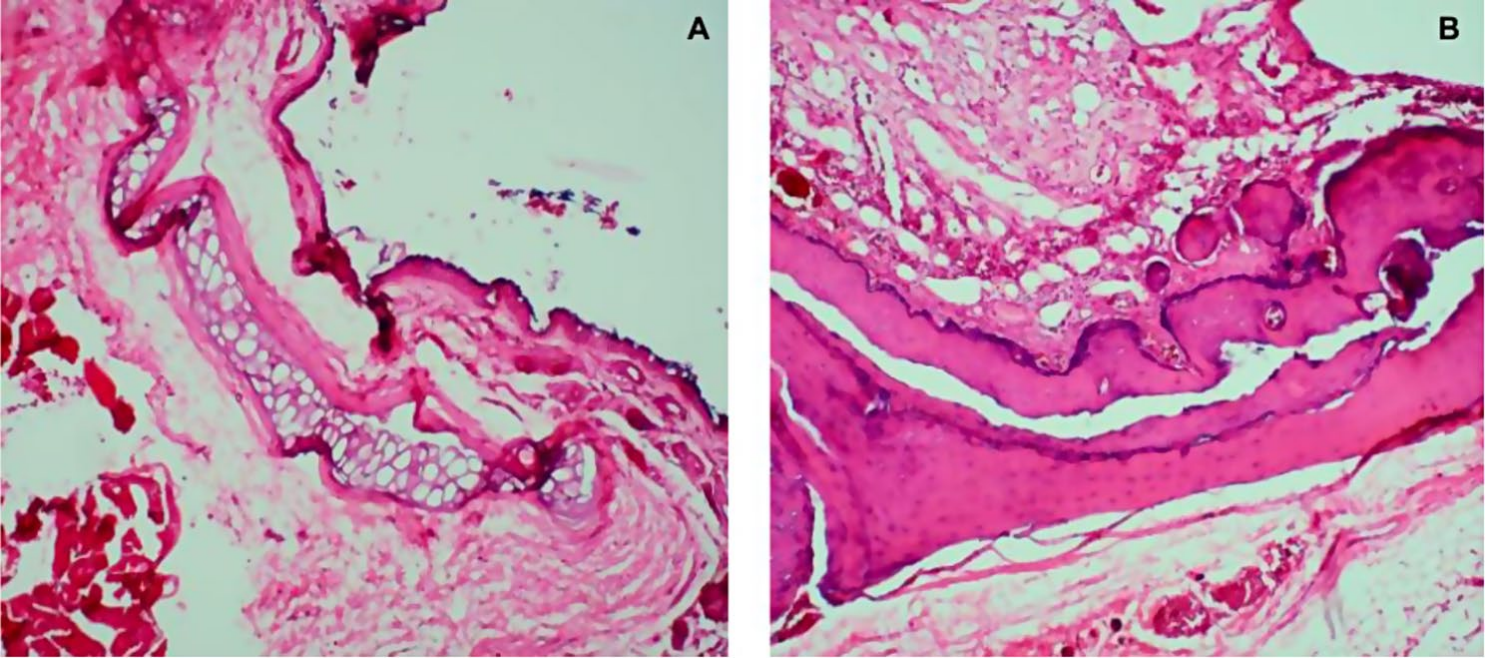
(**A**) Right Ear 2. Organ of Corti. There is an extensive focal area of epithelial damage in the inner ear, and an intense subepithelial inflammatory response affecting the bone. It is composed of a large number of degenerated neutrophils, lymphocytes, and a few macrophages, with edema, fibrosis, debris, and fibrin. (**B**) Left Ear 2. Organ of Corti. There is an extensive focal area of epithelial erosion in the inner ear, primarily affecting the endosteum. There is a zone of necrosis with abundant degenerated neutrophils, a smaller number of macrophages with foamy cytoplasm, abundant debris, fibrin, and moderate fibrosis

Inner ear: 100% of the specimens of Group 1 presented damage in the inner ear, ranging from minimal to moderate, including epithelial damage of the organ of Corti; lesion of the lamina propria and/or the bony labyrinth; edema, hemorrhage, inflammation, and fibrosis. When comparing Group 1 ears against Group 2 ears we found the difference in findings to be statistically significant with a *p* = 0.002 (Fig. 5).

**Table 2:** Evaluation of the tympanic membrane, tube biodegradation, and complications

	Study specimen			Pre-operative	Post-operative		
Number	Ear	Group	PLA insertion	Tympanic membrane	Tube degradation at 3 months	Tympanic membrane repair	Tympanic membrane
1	R	1	Yes	Retraction	Yes	Complete	Adhesive
1	L	1	Yes	Retraction	Yes	Complete	Adhesive
2	R	1	Yes	Normal	Yes	Complete	Normal
2	L	1	Yes	Normal	Yes	Complete	Adhesive
3	R	1	Yes	Retraction	Yes	Complete	Adhesive
3	L	2	No	Adhesive	N/A	N/A	Adhesive
4	R	2	No	Adhesive	N/A	N/A	Adhesive
4	L	2	No	Adhesive	N/A	N/A	Adhesive
5	R	2	No	Adhesive	N/A	N/A	Adhesive
5	L	2	No	Adhesive	N/A	N/A	Adhesive

R: right; L: left; N/A: not applicable

## Discussion

3D models are now being used as implants, opening up new options that are more patient-specific and cost-effective. This opens up a huge range of possibilities for 3D technology in the medical field [14, 15]. Although in Otolaryngology, there is a tendency to experiment with printing biological materials, in our study, we decided to experiment with the design and 3D printing of implants that can be easily manufactured at a lower cost. We used biocompatible materials that are easily accessible, with the aim that these same designs can, in the future, be shared by the medical community. We agree with McMillan et al. that although the literature reports successes in the fabrication of cartilage and bone structures for head and neck applications, the full potential of this technology has yet to be realized [16]. For the first time, we recognize that there is not yet a practical solution similar to what was presented by Zhang et al., where they designed a new artificial airway device based on many years of research experience in respiratory support therapy [17].

In our study, we initially utilized an implant that exhibited instability and low rigidity, resulting in collapse. However, following modification, it effectively fulfilled the desired functions.

The process of 3D printing patient-specific models is far from simple. According to Omari et al., it entails a complex series of steps, including preprocessing (such as capturing and editing image data for printing), processing, and postprocessing (involving the removal of excess impression material and sterilization) [18]. In addition, the choice of the most appropriate printing technology and materials also significantly affects the manufacturing process [19].

In a study conducted by Ensari et al., they applied a polylactic acid (PLA) film to the middle ear of guinea pigs. They found that it had no detrimental effects on hearing or middle ear mucosa compared to the non-intervened side [19]. However, a limitation of their study was the lack of measurement of DPOAES, which made it difficult to understand the effects of PLA on the inner ear. In our experiment, we observed a connection between PLA degradation and mild middle ear as well as moderate inner ear damage at different histological levels (RR 5, 95% CI 0.866–28.861, *p* = 0.010), which affected the production of DPOAEs. Therefore, the association between PLA and histological damage of the inner ear is confirmed by the absence of histological damage in the non-intervened ears, although it’s unclear if the damage is transient due to the short follow-up period.

It has been noted that traditional PLA 3D printers struggle to reproduce models smaller than 5 mm. Therefore, we suggest using a different type of material and 3D printer for such small implants. For example, Hirsch et al. [20] successfully reproduced an ossicular chain implant adapted to the anatomical circumstances of a defect in a cadaveric model. They replaced the incus (3 × 2 mm) with a 3D printed model using a resin printer with a precision of 25 μm, commonly used for dental stereolithographic models. However, since it was a cadaveric model, the effects of the resin on the inner ear were not known.

In our study, we observed the following post-implant changes in the middle ear: minimal to mild damage was observed in the epithelial layer of the tympanic membrane, as well as inflammatory damage in the middle ear in 80% of Group 1, in contrast to 0% of Group 2 in the inner ear: 100% of Group 1 specimens, presented damage in the inner ear, which ranged from minimal to moderate, among these: epithelial damage of the organ of Corti; lesion of the lamina propria and/or bony labyrinth; edema, hemorrhage, inflammation and fibrosis. Costa observed the temporal findings of 21 users where a chronic inflammatory process in the electrodes caused fibrosis and new bone formation; the inflammatory response was less intense around the electrodes closer to the most apical region of the cochlea [21].

At the first evaluation, at 3 months, remnants of PLA were observed in one ear, while in the rest there was no evidence of PLA with a completely repaired tympanic membrane. Throughout the observation period, no adverse events such as otorrhea, otorrhagia, granulomas, or other lesions occurred, and all specimens survived. It’s important to note that complications due to surgery at the time of implantation must also be considered. Lau presented the following complications of surgery in his study, which included blockage of the drainage with acute otitis media that required reoperation within six months in 3 out of 54 children who had the drainage placed [22].

It was found that 80% of the specimens exhibited middle ear pathology before the intervention, with 30% demonstrating atical retraction and 50% showing signs of glue ear. The remaining 20% showed no clinical alterations. Pre-intervention DPOAEs were present in 100% of Group 1 and 0% of Group 2. Absent emissions were indicative of adhesive tympanic membranes. Following the procedure, otoacoustic emissions were absent in all ears explored (100%).

We fully acknowledge the limitations of our study, such as the small sample size and the high prevalence of pre-existing middle ear pathology. Despite these challenges, we are confident in the importance of continuing our experimentation with 3D printing for implants. By strategically adjusting variables such as the biopolymer used, the quality, and the resolution of the 3D printer, we aim to improve the design and safety of the implant, while also identifying materials that are well tolerated at the otologic level.

## Conclusion

In our study, the design and 3D printing of implants achieved the desired functions. Among the complications, an association was found between PLA degradation and mild damage in the middle ear and moderate damage in the inner ear. In the evaluation corresponding to the observation period, there were no adverse events related to the implantation of the 3D model and all specimens were found to be surviving.

According to our results, since they showed small short-term improvements, we consider it necessary to deepen the research related to 3D printed models. These models offer the benefit of greater accessibility and the possibility of customizing implants according to the anatomical characteristics of the patient.

## Data Availability

No datasets were generated or analysed during the current study.

## References

[1] Venekamp RP, Mick P, Schilder AG, Nunez DA (2018). Grommets (ventilation tubes) for recurrent acute otitis media in children. Cochrane Database Syst Rev.

[2] Shaffer AD, Ford MD, Choi SS, Jabbour N (2017). The impact of Tympanostomy tubes on Speech and Language Development in Children with Cleft Palate. Otolaryngol Head Neck Surg.

[3] Gaddey HL, Wright MT, Nelson TN (2019). Otitis Media: Rapid evidence review. Am Fam Physician.

[4] Vanneste P, Page C (2019). Otitis media with effusion in children: pathophysiology, diagnosis, and treatment. A review. J Otol.

[5] Isaacson G (2020). Tympanostomy Tubes-A Visual Guide for the Young otolaryngologist. Ear Nose Throat J.

[6] Zhong N, Zhao X. 3D printing for clinical application in otorhinolaryngology. Eur Arch Otorhinolaryngol. 2017;274(12):4079–4089. doi: 10.1007/s00405-017-4743–0. Epub 2017 Sep 19. PMID: 28929219. 10.1007/s00405-017-4743-0 28929219

[7] Zoccali F, Colizza A, Cialente F, Di Stadio A, La Mantia I, Hanna C, Minni A, Ralli M, Greco A, de Vincentiis M (2022). 3D Printing in Otolaryngology surgery: descriptive review of literature to define the state of the art. Healthc (Basel).

[8] Hong CJ, Giannopoulos AA, Hong BY, Witterick IJ, Irish JC, Lee J, Vescan A, Mitsouras D, Dang W, Campisi P, de Almeida JR, Monteiro E (2019). Clinical applications of three-dimensional printing in otolaryngology-head and neck surgery: a systematic review. Laryngoscope.

[9] Murphy SV, De Coppi P, Atala A (2020). Opportunities and challenges of translational 3D bioprinting. Nat Biomed Eng.

[10] Lawrence R, McGowan P, Daniel M (2017). An all-in-one tympanostomy tube insertion device: results of usability testing in fourteen cadaveric and twelve plastic model ears. Clin Otolaryngol.

[11] Whelan RL, Maguire RC. Tympanostomy Tube Innovation: Advances in Device Material, Design, and Office-Based Technology. Ear Nose Throat J. 2020;99(1_suppl):48S–50S. doi: 10.1177/0145561320924910. Epub 2020 Jun 2. PMID: 32484409. 10.1177/0145561320924910 32484409

[12] Jones A, Walter S, M. Shortages of Care and Medical Devices Affecting the Pediatric Patient Population. : CADTH Horizon Scan [Internet]. Ottawa (ON): Canadian Agency for Drugs and Technologies in Health; 2023 Aug. Report No.: EH0116. PMID: 37934838. 37934838

[13] You P, Bartellas M. Three-dimensional Printing in Pediatric Otolaryngology. Otolaryngol Clin North Am. 2022;55(6):1243–1251. 10.1016/j.otc.2022.07.013. PMID: 36371138. 10.1016/j.otc.2022.07.013 36371138

[14] Omari A, Frendø M, Sørensen MS, Andersen SAW, Frithioff A (2022). The cutting edge of customized surgery: 3D-printed models for patient-specific interventions in otology and auricular management-a systematic review. Eur Arch Otorhinolaryngol.

[15] Ensari N, Tutar H, Ekinci O, Ugur MB, Bayazıt YA, Gokdogan C (2015). Effects of polylactic acid film on middle ear mucosa and cochlear function in Guinea pigs. Eur Arch Oto-Rhino-Laryngology.

[16] Hirsch JD, Vincent RL, Eisenman DJ (2017). Surgical reconstruction of the ossicular chain with custom 3D printed ossicular prosthesis. 3D Print Med.

[17] Costa LBAD, Vicente LC, Silva LTDN, Alvarenga KF, Salgado MH, Costa OA, Brito R. Analysis of aided thresholds in children who have undergone cochlear reimplantation: a ten-year follow-up. Codas. 2023;35(6):e20210293. Portuguese, English. 10.1590/2317-1782/20232021293pt. PMID: 37909539. 10.1590/2317-1782/20232021293en PMC10702709 37909539

[18] Lau L, Mick P, Nunez DA (2018). WITHDRAWN: grommets (ventilation tubes) for recurrent acute otitis media in children. Cochrane Database Syst Rev.

[19] Zhong N, Zhao X (2017). 3D printing for clinical application in otorhinolaryngology. Eur Arch Oto-Rhino-Laryngology.

[20] Canzi P, Magnetto M, Marconi S, Morbini P, Mauramati S, Aprile F (2018). New frontiers and emerging applications of 3D printing in ENT surgery: a systematic review of the literature. Acta Otorhinolaryngol Ital.

[21] McMillan A, McMillan N, Gupta N, Kanotra SP, Salem AK. 3D Bioprinting in Otolaryngology: A Review. Adv Healthc Mater. 2023;12(19):e2203268. doi: 10.1002/adhm.202203268. Epub 2023 Mar 31. PMID: 36921327; PMCID: PMC10502192. 10.1002/adhm.202203268 PMC10502192 36921327

[22] Zhang J, Li H. [A new type of artificial airway sealer used between artificial airway and ventilator pipeline]. Zhonghua Wei Zhong Bing Ji Jiu Yi Xue. 2023;35(9):991–994. Chinese. 10.3760/cma.j.cn121430-20230310-00168. PMID: 37803961. 10.3760/cma.j.cn121430-20230310-00168 37803961

